# Predictive value of facial motor–evoked potential and electromyography for facial motor function in vestibular schwannoma surgery

**DOI:** 10.1007/s00701-024-05927-0

**Published:** 2024-01-19

**Authors:** Kathrin Machetanz, Martin Roegele, Marina Liebsch, Linda Oberle, Eliane Weinbrenner, Mykola Gorbachuk, Sophie S. Wang, Marcos Tatagiba, Georgios Naros

**Affiliations:** Department of Neurosurgery and Neurotechnology, Eberhard Karls University, Hoppe-Seyler-Straße 3, 72076 Tuebingen, Germany

**Keywords:** Vestibular schwannoma, Facial palsy, Motor-evoked potential, Electromyography, House-Brackmann score

## Abstract

**Purpose:**

Intraoperative neuromonitoring (IONM) aims to preserve facial nerve (FN) function during vestibular schwannoma (VS) surgery. However, current techniques such as facial nerve motor evoked potentials (FNMEP) or electromyography (fEMG) alone are limited in predicting postoperative facial palsy (FP). The objective of this study was to analyze a compound fEMG/FNMEP approach.

**Methods:**

Intraoperative FNMEP amplitude and the occurrence of fEMG-based A-trains were prospectively determined for the orbicularis oris (ORI) and oculi (OCU) muscle in 322 VS patients. Sensitivity and specificity of techniques to predict postoperative FN function were calculated. Confounding factors as tumor size, volume of intracranial air, or IONM duration were analyzed.

**Results:**

A relevant immediate postoperative FP was captured in 105/322 patients with a significant higher risk in large VS. While fEMG demonstrated a high sensitivity (77% and 86% immediately and 15 month postoperative, respectively) for identifying relevant FP, specificity was low. In contrast, FNMEP have a significantly higher specificity of 80.8% for predicting postoperative FP, whereas the sensitivity is low. A retrospective combination of techniques demonstrated still an incorrect prediction of FP in ~ 1/3 of patients.

**Conclusions:**

FNMEP and fEMG differ in sensitivity and specificity to predict postoperative FP. Although a combination of IONM techniques during VS surgery may improve prediction of FN function, current techniques are still inaccurate. Further development is necessary to improve IONM approaches for FP prediction.

## Introduction

Vestibular schwannomas (VS) account for ~80% of all tumors of the cerebellopontine angle, constituting the most common entity in this location [13, 23, 38]. Due to the anatomic relation to the internal auditory canal and the traversing vestibulocochlear (VIII) and facial (VII) nerve, surgical resection of VS entails the risk of hearing loss and facial palsy (FP). Continuous intraoperative neuromonitoring (IONM) and advances in surgical techniques have improved anatomical facial nerve (FN) preservation. However, literature suggests that severe FP still occurs in 7–15% of the cases, with tumor size as the most important predictive factor [15] and potential reduction of physical and mental health as clinical consequence [21].

IONM is accepted as a general standard in VS surgery and is intended to provide anatomic identification of the FN, protect against potentially damaging events causing functional FN deterioration, and prognostically predict postoperative FN function [31]. However, a standardized IONM approach is lacking. Depending on the center facial electromyography (fEMG) [27, 28, 33], facial nerve motor–evoked potentials (FNMEP) via transcranial electrical stimulation (TES) [1, 3, 4, 22], or a combination of techniques are used for monitoring. fEMG can be performed continuously, but quantitative analysis of pathological A-trains during surgery is difficult. Even with techniques for automated processing of A-train duration, previous studies yield positive predictive values of only ~64% for predicting FN outcome [29, 30]. FNMEP monitoring allows simple analyses of final-to-baseline amplitudes and can be performed frequently but not continuously like the fEMG. Positive predictive values for FNMEP also range only between 53 and 61% [31].

In conclusion, previous studies have demonstrated limited predictive power of monitoring techniques. The analyses, however, mostly enrolled only a small number of patients (< 100 persons). In addition, an evaluation that combines techniques and systematically investigates confounding factors is lacking. This study aims to describe the FN monitoring technique used in our tertiary neurosurgical center and analyses the predictive power of methods in 322 patients.

## Methods

### Clinical data

This retrospective analysis of prospectively collected data enrolled 322 patients (49.4 ± 13.1 years, 169 female) who underwent neurosurgical resection of a VS at the Department of Neurosurgery of the University of Tuebingen between 2011 and 2016. Decision for resection was based on patients’ age, tumor size, VS-associated symptoms and patients’ therapeutic preference. Patients with prior (radio-)surgery of the VS and preoperative facial nerve palsy were excluded. Tumor extent was graded according to the Koos classification (Koos 1: purely intrameatal, Koos 2: intra- and extrameatal, Koos 3: filling the cerebellopontine cistern, Koos 4: compressing or shifting the brainstem). Intraoperative positioning was dependent on tumor size. The majority of patients with Koos 1 and 2 tumors were operated in supine position, whereas Koos 3 and 4 tumors were operated in semi-sitting position. The study was approved by the local ethics committee of the Eberhard Karls University Tuebingen and performed in accordance with the Declaration of Helsinki. Patients‘ characteristics are summarized in Table 1.

**Table 1:** Patient characteristics

	Overall (*n* = 322)	Postoperative HB < III° (*n* = 217)	Postoperative HB ≥ III° (*n* = 105)	
Age (years)	49.4 ± 13.1	48.5 ± 12.2	51.2 ± 14.9	*H* = 3.95 ***p*** **= 0.047**
Gender				*X*^2^ = 0.47; *p* = 0.491
Female	169 (52.5%)	111 (51.2%)	58 (55.2%)	
Male	153 (47.5%)	106 (48.8%)	47 (44.8%)	
Tumor size				*X*^2^ = 34.76; *p* < 0.001
Koos 1	12 (3.7%)	10 (4.6%)	2 (1.9%)	
Koos 2	71 (22.0%)	61 (28.1%)	10 (9.5%)	
Koos 3	144 (44.7%)	103 (47.5%)	41 (39.0%)	
Koos 4	95 (29.5%)	43 (19.8%)	52 (49.5%)	
Tumor side				*X*^2^ = 0.07; *p* = 0.791
Left	156 (48.4%)	102 (47%)	51 (48.6%)	
Right	166 (51.6%)	115 (53%)	54 (51.4%)	
Positioning				*X*^2^ = 8.53; ***p* = 0.003**
Supine	60 (18.6%)	50 (23%)	10 (9.5%)	
Semi-sitting	262 (81.4%)	167 (77%)	95 (90.5%)	
Facial nerve outcome (HB) immediately postop				
I	149 (46.3%)	149 (68.7%)	0 (0%)	
II	68 (21.1%)	68 (31.3%)	0 (0%)	
III	35 (10.9%)	0 (0%)	35 (33.3%)	
IV	52 (16.1%)	0 (0%)	52 (49.5%)	
V	18 (5.6%)	0 (0%)	18 (17.1%)	
VI	0 (0%)	0 (0%)	0 (0%)	
Postoperative pneumocephalus (PP; ml)	33.6 ± 33.0	35.8 ± 34.4	29.1 ± 29.6	*H* = 1.15 *p* = 0.284

*p* < 0.05 (bold) indicate significant differences between postoperative groups HB < III° vs. HB ≥ III°

*HB* House-Brackmann score

### Intraoperative neuromonitoring (IONM)

Electrophysiological measurements during the surgery were performed by experienced electrophysiologists using an Endeavor monitoring unit (Endeavor, Viasys Healthcare, Madison, WI, USA). In addition to continuously electromyography (fEMG) of the facial nerve and facial motor evoked potentials (FNMEP), motor (MEP) and somatosensory evoked potentials (SEP) of the hands and legs were monitored, as described in detail previously [1, 4]. In patients with tumors reaching the lower cranial nerves, EMG of the glossopharyngeal, vagal, accessory, and hypoglossal nerve was measured as well. Furthermore, brainstem auditory evoked potentials (BAEP) of the tumor side were monitored in patients with residual hearing function.

**Fig. 1:**
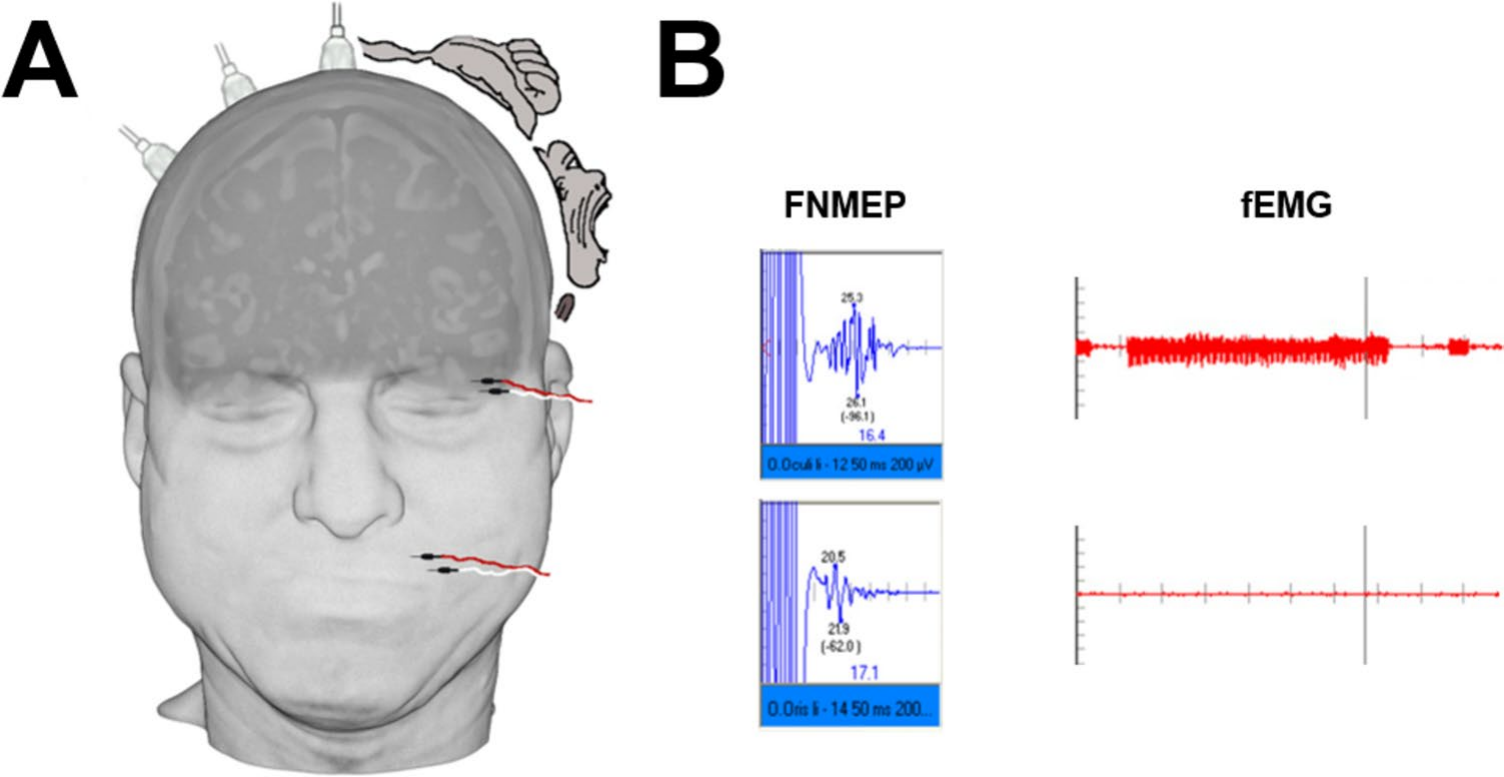
Schematic illustration of **A** the electrode positioning with corkscrew electrodes in Cz, C2 and C4 as well as needle electrodes in the orbicularis oris and oculi muscle and **B** the facial nerve motor evoked potentials (FNMEP) as well as facial nerve electromyography (fEMG) demonstrating A-trains in the orbicularis oculi muscle.

Transcranial electrical stimulation (TES) for FNMEP monitoring was performed using corkscrew-like electrodes, which were positioned at Cz and C3 or C4 according to the international 10/20 system for left or right sided stimulation, respectively (Fig. 1). Stimulation was always applied using one, three, or five rectangular pulses, ranging from 200 to 400 V with a 500-μs pulse duration and an interstimulus interval (ISI) of 2 ms. Facial potentials were recorded from needles placed in the orbicularis oculi (OCU) and oris (ORI) muscles of the affected and healthy side (Fig. 1). Stimulations with one and five pulses were matched to ensure that the facial nerve was not stimulated extracranially. The abductor pollicis brevis (APB) muscle was used as a control of the general motor cortical excitability. MEPs of the hands and legs were stimulated by C1 and C2. TES was performed intermittently with SEP and BAEP recordings. The surgeon was always informed prior to stimulation, as TES may cause undesirable movements. For facial nerve function estimation and further analyses, the best response before dural opening was used as baseline value. Final values were defined at the moment of dural closure, and the final-to-baseline amplitude (FBR) was estimated. FNMEP amplitude was defined as the voltage between the maximum positive and negative deflection of the waveforms. FNMEP latency was defined as the time from stimulus onset to the first wave deflection.

For the fEMG, the same needle electrodes in the OCU and ORI as for FNMEP were used. The occurrence of A-trains, which are considered pathological [33], was detected and reported to the surgeon.

### Facial nerve function

Facial nerve function was categorized according to the House–Brackmann (HB) grading system within 1 week after the surgery (immediately postoperative) as well as 15 months after surgery by experienced neurosurgeons. HB grades I and II were classified as satisfactory, while HB grades III to VI were assessed as unsatisfactory.

### Statistics

Statistical tests were performed using the SPSS (IBM SPSS Statistics for Windows, Version 26.0. Armonk, NY: IBM Corp.). Sensitivity, specificity, and positive and negative predictive values of FNMEP and fEMG to predict the FN outcome were determined by differentiating the occurrence of A-trains (yes/no) and using various amplitude FBR thresholds (0.5, 0.6, 0.7) in FNMEP (Table 2). The sensitivity (1) informs us how often the monitoring test (e.g., A-train yes or ORI-MEP <0.6) detects a facial palsy when postoperative a relevant facial palsy is apparent (true positive rate, TPR). The specificity (2) indicates how often the monitoring test detects “no palsy” while there is no postoperative facial palsy (true negative rate, TNR). The positive (PPV) predictive values are the proportions of patients with positive test results (e.g., A-train yes or ORI-MEP <0.6) who already have a facial palsy (3), while the negative predictive value (NPV) is the proportion of the cases giving negative test results who are already healthy (4) (Table 2)

**Table 2:** Exemplary contingency table for the FNMEP test

		Facial palsy		
		YES (+)	NO (−)	
ORI-MEP < 0.6	YES (+)	True positive (TPR)	False positive (FPR)	$PPV=\frac{TPR}{TPR+ FPR}$ (3)
	NO (−)	False negative (FNR)	True negative (TNR)	$NPV=\frac{TNR}{FNR+ TNR}$ (4)
		$\textrm{Sensitivity}=\frac{TPR}{TPR+ FNR}$ (1)	$\textrm{Specificity}=\frac{TNR}{\ FPR+ TNR}$ (2)	

Group differences of clinical characteristics (e.g., patients‘ age, tumor size) in patients with and without satisfactory FN outcome as well as true and false classified patients were determined by chi-squared or Kruskal-Wallis tests. Correlation analyses were performed by Spearman’s correlation. Statistical significance was considered at *p* < 0.05 for each statistical test.

## Results

### Patient cohort and surgical results

Continuous fEMG and FNMEP monitoring was performed in 322 patients (49.4 ± 13.1 years, 169 female) during VS surgery in semi-sitting (262/322) or supine (60/322) position (Table 1). In 83/322 (25.8%) patients, the VS corresponded to a tumor size Koos 1 or 2 and in 239/322 (74.2%) to Koos 3 or 4. Patients with preoperative facial nerve paresis were excluded. A total of 105 patients (32.6%) suffered from relevant facial nerve paresis (≥ HB III°) immediately postoperatively. At 15-month follow-up, most of them improved to HB I-II°, and 22/281 (6.8%) remained with HB ≥ III°. Forty-one patients (15 with HB I–II° and 36 with HB (≥ HB III° immediately postoperative) did not present for follow-up examination. Tumor size correlated significantly with the grade of postoperative facial palsy (*r* = 0.42, *p* < 0.001; Spearman’s). Only 12/83 (15%) patients with tumor size Koos 1 or 2 had relevant facial palsy, while 93/239 (39%) patients with tumor size Koos 3 or 4 suffered from postoperative facial nerve paresis ≥ HB III° immediately after surgery. This difference was also evident for positioning as Koos 1/2 mostly were treated in supine position, while Koos 3/4 VS are operated in semi-sitting position.

### Predictive value of facial nerve EMG and MEP

**Fig. 2:**
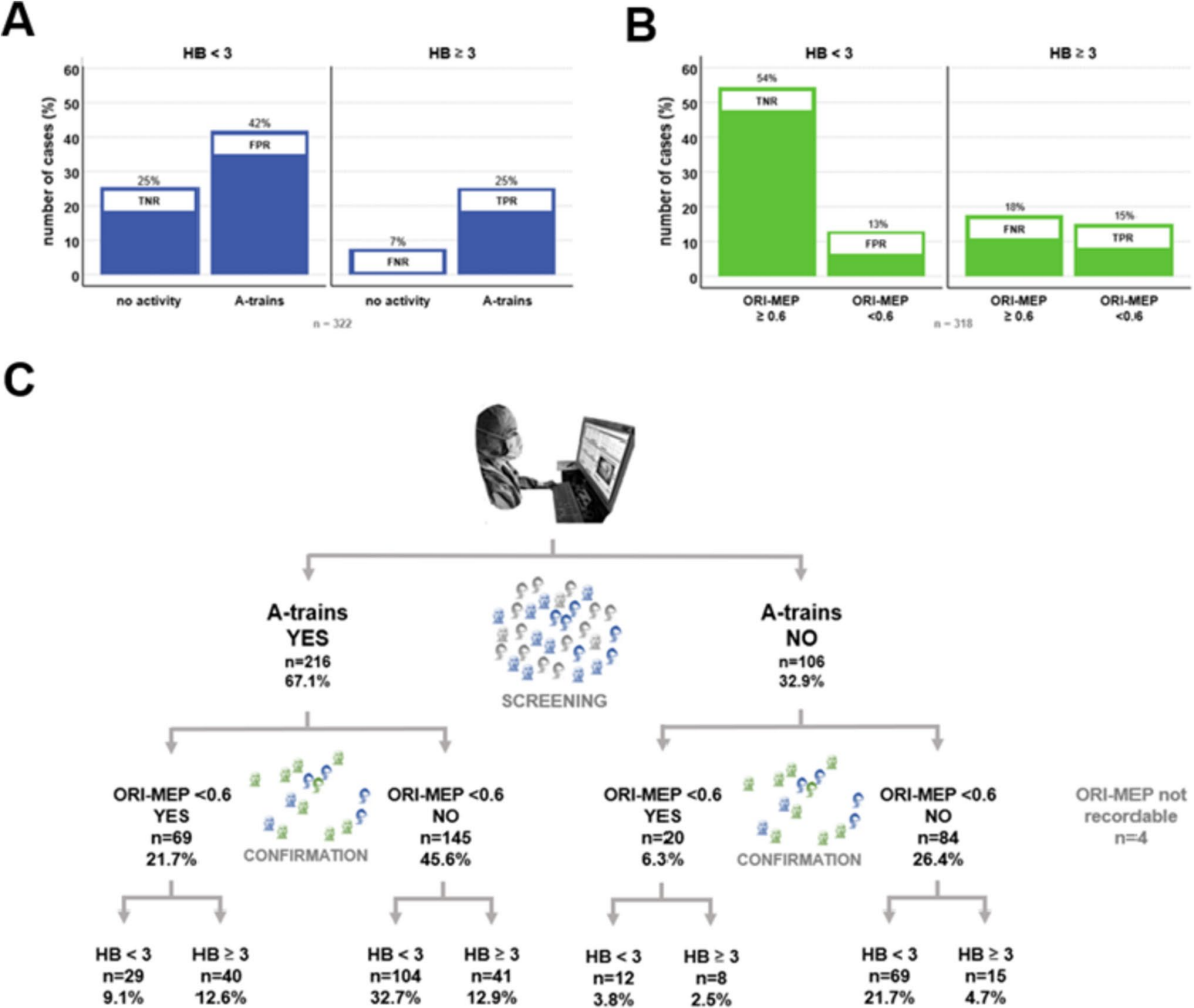
Intraoperative facial nerve monitoring by electromyography (fEMG) and motor evoked potentials (FNMEP). **A** and **B** demonstrate separately the predictive values of the fEMG and FNMEP technique. **C** presents a combined monitoring approach with fEMG as screening and FNMEP as confirmatory test. FNR, false negative rate; FPR, false positive rate; HB, House-Brackmann score; ORI, orbicularis oris; TNR, true negative rate; TPR, true positive rate

In a total of 216/322 (67.1%) patients, A-trains were detected during fEMG monitoring. Sensitivity for detecting a facial palsy ≥ HB III° was 77.1% immediately postoperative (days 1–6) and 86.4% 15 months postoperative. However, there was a low specificity of 37.8% for A-trains predicting facial nerve outcome (Table 3). These values resulted in a total of 163/322 (50.6%) correct classified patients (TP + TN), 135/322 (41.9%) false positive (FPR), and 24/322 (7.4%) false negative (FNR) detected patients by fEMG (Fig. 2A). There were no differences between correct classified patients (TPR + TNR) compared to FPR and FNR groups regarding age, gender, tumor size and side, positioning, or volume of postoperative pneumocephalus. In contrast, FNMEP final-to-baseline ratios showed overall a high specificity, but low sensitivity for predicting a relevant facial palsy ≥ HB III° (Table 3). FNMEP of the orbicularis oris muscle (ORI-MEP) had a significant higher predictive power than FNMEP of the orbicularis oculi (OCU-MEP). Hence, further FNMEP analysis and descriptions refer to the ORI-MEP with a 0.6-FBR. A total of 221/318 (69.5%) patients were classified correctly by these 0.6-FBR ORI-MEP, while the facial nerve outcome was false positive and negative predicted in 41/318 (12.9%) and 56/318 (17.6%) cases. In four patients, the ORI-MEP was not recordable.

**Table 3:** Predicting significant facial nerve deterioration

	Postoperative days 1–6				Postoperative 15 months			
	*sens*	*spec*	*PPV*	*NPV*	*sens*	*spec*	*PPV*	*NPV*
ORI-MEP TH 0.5	37.5%	86.9%	58.2%	74.1%	50%	85.9%	23.4%	95.2%
ORI-MEP TH 0.6	46.2%	80.8%	53.9%	75.5%	59.1%	78.9%	19.4%	95.7%
ORI-MEP TH 0.7	54.8%	73.4%	50.0%	77.0%	71.9%	77.3%	19.1%	97.4%
OCU-MEP TH 0.5	30.4%	86.4%	51.7%	72.3%	31.8 5	84.7%	15.2%	93.5%
OCU-MEP TH 0.6	38.2%	82.2%	50.6%	73.6%	40.9%	80.4%	15.3%	94.0%
OCU-MEP TH 0.7	46.1%	75.7%	47.5%	74.7%	50.05	73.7%	14.1%	94.5%
fEMG/A-trains	77.1%	37.8%	37.5%	77.4%	86.4%	36.3%	7.8%	96.9%

*fEMG* facial electromyography, *MEP* motor-evoked potential, *NPV* negative predictive value, *OCU* orbicularis oculi, *ORI* orbicularis oris, *PPV* positive predictive value, *sens* sensitivity, *spec* specificity, *TH* threshold

### A combined EMG/MEP-approach

Retrospectively, we analyzed a combined approach of fEMG and FNMEP monitoring based on the presented data (Fig. 2C). Diagnostic tests with a high sensitivity are well suited as screening test. Therefore, fEMG was used as screening test and only the 216 patients in whom intraoperative A-trains were detected were examined by 0.6-FBR ORI-MEP (confirmatory test). In two of the patients, no ORI-MEP was recordable. A total of 69/214 (32.2%) patients had a decline in ORI-MEP <0.6, whereas 145/214 (67.8%) patients had no decrease below the threshold. Forty out of the 69 (58%) patients with an ORI-MEP <0.6 showed a relevant postoperative facial paresis immediately postoperative and where therefore classified as TP. In contrast, 104/145 (71.7%) patients were classified true negative resulting in 144/214 (67%) correctly classified (TPR + TNR) cases in this cohort. However, there were still 41/145 (28.3%) or 41/214 (19.2%) which were considered false negative (FNR). To improve the combined approach in future, it should be therefore the main effort to better differentiate between true and false negative FNMEP in patients with detected A-trains and define relevant factors affecting measurements. However, while there was a trend of higher weights in the false negative group (*H* = 3.43, *p* = 0.064), we could not detect significant differences between the TNR and FNR group in our data (Table 4).

**Table 4:** FNMEP in patients with A-trains

	Overall (*n* = 214)	TNR (*n* = 104)	FNR (*n* = 41)	
Age (years)	49.8 ± 13.6	48.4 ±10.9	51.8 ± 17	*H* = 2.95 *p* = 0.086
Gender				*X*^2^ = 0.85; *p* = 0.358
Female	121 (56.6%)	57 (55%)	19 (46%)	
Male	93 (43.5%)	47 (45%)	22 (54%)	
Weight (kg)	75.1 ± 16.8	75.3 ± 16.4	81.2 ± 19	*H* = 3.43 *p* = 0.064
Size (cm)	172.0 ± 9.3	172.2 ± 9.2	173.5 ± 9	*H* = 0.49 *p* = 0.483
Tumor size				*X*^2^ = 3.18; *p* = 0.074
Koos 1 + 2	47 (22%)	30 (29%)	6 (15%)	
Koos 3 + 4	167 (78%)	74 (71%)	35 (85%)	
Tumor side				*X*^2^ = 0.01; *p* = 0.938
Left	107 (50%)	54 (52%)	21 (51%)	
Right	107 (50%)	50 (48%)	20 (49%)	
Positioning				*X*^2^ = 1.56; *p* = 0.212
Supine	36 (16.8%)	22 (21%)	5 (12%)	
Semi-sitting	178 (83.2%)	82 (79%)	36 (88%)	
IOM duration (min)	270.1 ± 74.0	263.2 ± 77.7	274.7 ± 78	*H* = 0.71 *p* = 0.398
Postoperative pneumocephalus (PP; ml)	33.6 ± 32.6	34.4 ± 31.5	29.4 ± 32	*H* = 0.62 *p* = 0.429

FNMEP in patients with A-trains demonstrates no significant clinical differences of true negative (TPR) and false negative (FNR) 0.6-FBR of ORI-MEP (facial nerve evaluation immediately postoperative)

### Impact of intracranial air on monitoring results

Since the previous analyses could not demonstrate any clear factors influencing the “false prediction,”, we further analyzed the influence of postoperative pneumocephalus (PP) in facial nerve monitoring, since an influence of PP on electrophysiological measurements has been postulated repeatedly. The mean intracranial air of the total cohort was 33.6 ± 33 ml. Patients operated in a semi-sitting position had a significantly larger amount of intracranial air than those operated supine (41.0 ± 32 ml and 1.0 ± 2 ml) (*H* = 132.86, *p* < 0.001). To analyze the influence of intracranial air on FNMEP, a subcohort analysis of patients who did not have relevant facial nerve palsy (Table 1) was performed, as there was no change in FNMEP due to surgical lesions in these patients. In this cohort of 217 patients (111 female), mean intracranial air was 35.8 ± 24 ml. There was no significant correlation of the volume of intracranial air to the 0.6-FBR ORI-MEP (*r* = −0.004, *p* = 0.957; Spearman’s). However, the final-to-baseline MEP amplitude ratio of the ipsilateral hand correlated significantly negative with the volume of intracranial air (*r* = −0.36; *p* < 0.001; Spearman’s) predisposing the hand MEP for estimating the amount of intracranial air.

## Discussion

Intraoperative facial nerve monitoring is considered as a gold standard in VS surgery to preserve facial integrity [39]. The occurrence of A-trains is regarded as a warning criterion in fEMG monitoring (i.e., train-time criterion) [29, 30], while a reduction in amplitude of more than 40–50% is assessed as critical in FNMEP (i.e., final-to-baseline ratio, FBR) [5, 7, 8, 14, 41]. However, quantitative analysis of pathological A-trains with differentiation to other EMG patterns during surgery is difficult [29, 30, 33]. In addition, the fEMG has a high sensitivity with low specificity for predicting significant FN deterioration. In contrast, FNMEP has a better specificity with low sensitivity to predict significant FN deterioration [1, 7, 14, 41]. The present study analyzed the prognostic value of a combination of both techniques in a large cohort of >300 VS patients.

Our results demonstrate that facial outcome prediction remains incorrect in nearly one-third of patients by combining FNMEP and fEMG as presented. The subsequent purpose of further investigations has to be an improvement of this rating. However, there is little interpretive margin for the monitoring assistant during surgery with homogeneous, but false results of both techniques. Although monitoring methods in these cases provide an incorrect prediction, the homogeneous rating of different techniques suggests that the measurement works properly from a technical point of view. Other factors (e.g. anesthetics, blood pressure, temperature) may influence the FN outcome. Overall, 4.7% of our patient cohort were detected false negative by fEMG and FNMEP. In these cases, the facial nerve may be unaffected during surgery, while there are adverse factors immediately after the monitoring or surgery phase deteriorating the FN — e.g., fluctuations of blood pressure with hypotonia, which can lead to vascular events. This hypothesis is supported by the fact that various studies have demonstrated the neuroprotective value of vasoactive drugs (e.g., nimodipine) [29–31]. It has been shown that the occurrence of delayed facial palsy was more likely after the discontinuation of a nimodipine therapy and that patients with postoperative facial palsy had a better long-term outcome with nimodipine therapy [29, 31]. Thus, good blood pressure control and the administration of vasoactive drugs should be aspired in postoperative management. However, these factors cannot be controlled by the monitoring assistant intraoperatively. In this situation, the correct interpretation of discrepant results with e.g. true-positive A-trains in the screening test, but false-negative, stable FNMEP-amplitudes is crucial. The lower sensitivity of FNMEPs, which is responsible for this, can be improved by re-defining the critical threshold. Whereas previous studies mostly considered an amplitude reduction of 50% as relevant [5, 22, 41], our results — in line with other recent studies [7, 14] — demonstrated a higher predictive value of FN outcome for a threshold of 0.6 (i.e., an amplitude reduction of 40%). Furthermore, alternative FNMEP analysis techniques than the FBR analysis — as e.g., event-to-baseline amplitude or a minimal-to-baseline ratio — may can improve the sensitivity [14]. Alternatively to the improvement of fEMG and FNMEP analyses, an evoked facial nerve EMG within the cerebellopontine angle [6, 42] or the application of direct nerve stimulation (DNS) [11, 25, 35] as further monitoring technique is also possible. DNS enables anatomic identification of the FN. However, usually, it can only be applied intermittently, as the stimulation probe has to be used selective by the surgeon. The implementation of a combined surgical suction-and-mapping probe can partially resolve this problem by a continuous dynamic mapping [36]. However, as in VS surgery often a bimanual preparation technique [10, 40] is used, even such a probe cannot be used continuously. Furthermore, prognostic analyses based on proximal-to-distal amplitude ratios in DNS could demonstrate positive predictive values of only ~46% [35].

The stepwise monitoring approach (i.e., 1: fEMG as screening test, 2: FNMEP as confirmatory test) enables to reduce the high number of false positive detected A-trains by the FNMEP method. Nevertheless, in our study, 9% of patients were detected false positive by both techniques. Consequently, the false positive rate of the FNMEPs is also a matter of interest in facial nerve monitoring. It might be associated to intracranial air, patient temperature, depth of anesthesia, and fluctuations of the impedances or the excitability: The volume of postoperative pneumocephalus is often postulated as a source of error for false positive deterioration in motor evoked potentials. However, previous studies demonstrated inconclusive results with and without PP-associated MEP-changes [2, 17, 32]. Our results showed a deterioration of the hand MEP, whereas no air-dependent deterioration of the FNMEP could be demonstrated. This fact may be explained by the intraoperative positioning of the patient as well as the positioning of the electrodes: in semi-sitting position, the amount of pneumocephalus is usually higher than in supine position [18]. Since the air usually accumulates at the highest point, it can be assumed that the air is more likely to be present under electrodes C1 and C2 than under electrodes C3 and C4, which are used for measuring the FNMEP (Fig. 1).

Experimental studies in rats demonstrated a decrease of spinal motor evoked potentials in hypothermia [26]. In contrast, hyperthermia in combination with high impedances may increase noise and decrease the statistical power during electrophysiological monitoring [16]. While the influence of intraoperative impedance changes could be reduced by a current-regulated stimulation, spontaneous fluctuations of the cortical excitability with the consequence of a high intra-subject variability of the MEP amplitude [9, 19] could be addressed by alternative FNMEP analyses techniques than the FBR analysis. Previous studies considered the application of an event-to-baseline amplitude or a minimal-to-baseline ratio including a recovery value [14]. Here, the lowest amplitude value during the surgery is measured and related to the baseline amplitude as well as to the amplitude at the end of the surgery. Another method used is the threshold level method, in which the difference in stimulation intensity is evaluated while the response amplitude is kept constant by increasing the stimulation intensity [34, 37]. Greve et al. [12] used the non-lesional side as an additional evaluation criterion to the lesioned side. Finally, time frequency analysis, which has recently shown promising results in the analysis of motor potentials evoked by TMS [19, 20, 24], suggests a promising further development of facial nerve monitoring.

### Limitations

While the number of patients analyzed and the use of combined monitoring techniques is the strength of the results presented, the retrospective evaluation is the major weakness. Especially in fEMG analyses, we could only differentiate between the occurrence of A-trains vs. the non-occurrence. A statement about the duration of the A-trains could not be made. Furthermore, in FNMEP analysis, only the FBR was documented, while event-to-baseline amplitude or a minimal-to-baseline ratio is lacking. Finally, we did not evaluate individual anesthetic dosages (e.g., analgesics, muscle relaxants) or body temperatures, which may have influenced the measurement. However, all patients were operated in a highly standardized surgical and anesthesiological setting, which has been described in previous studies [1]. Further prospective studies, which also investigate innovative analysis techniques such as time-frequency analysis, should address these limitations in future.

## Conclusion

The combination of fMEP/FNMEP for monitoring the facial nerve still leads to significant false predictions of postoperative facial outcome. Thus, further development of the analysis techniques and parameters is necessary to better predict the facial nerve outcome and thus to guarantee the function-oncological outcome by an extensive resection on the one hand and a good facial function on the other hand.

## Data Availability

Data can be provided on reasonable request.

## Abbreviations

AUC: area under the curve
BAEP: brainstem auditory evoked potential
DNS: direct nerve stimulation
FBR: final-to-baseline ratio
fEMG: facial electromyography
FN: facial nerve
FNMEP: facial nerve motor evoked potentials
FNR: false negative rate
FP: facial palsy
FPR: false positive rate
NPV: negative predictive value
HB: House-Brackmann score
IONM: intraoperative neuromonitoring
MEP: motor evoked potential
OCU: orbicularis oculi muscle
ORI: orbicularis oris muscle
PP: postoperative pneumocephalus
PPV: positive predictive value
TES: transcranial electrical stimulation
TNR: true negative rate
TPR: true positive rate
VS: vestibular schwannoma
